# Myopericarditis complicated by pulmonary embolism in an immunocompetent patient with acute cytomegalovirus infection: a case report

**DOI:** 10.1186/1756-0500-7-193

**Published:** 2014-03-28

**Authors:** Yves Marie Vandamme, Alexandra Ducancelle, Loïc Biere, Nathalie Viot, Frédéric Rouleau, Valérie Delbos, Pierre Abgueguen

**Affiliations:** 1 Department of Infectious Diseases and Internal Medicine, Centre Hospitalier Universitaire d’Angers, 4 rue Larrey, 49933 Angers, Cedex 9, France; 2 Department of Bacteriology and Virology, Centre Hospitalier Universitaire d’Angers, 4 rue Larrey, 49933 Angers, Cedex 9, France; 3 Department of Cardiology, Centre Hospitalier Universitaire d’Angers, 4 rue Larrey, 49933 Angers, Cedex 9, France; 4 Department of Infectious and Tropical Diseases, CHU Angers, 4 rue Larrey, 49933 Angers, Cedex 9, France

**Keywords:** Cytomegalovirus, Immunocompetent patient, Myopericarditis, Pulmonary embolism, Thrombosis

## Abstract

**Background:**

Primary acute cytomegalovirus infection in immunocompetent patients is common worldwide. Infection is most often asymptomatic or occurs sub-clinically with a self-limited mononucleosis-like syndrome. More rarely, the infection may lead to severe organ complications with pneumonia, myocarditis, pericarditis, colitis and hemolytic anemia. Recent cases of cytomegalovirus-associated thrombosis have also been reported sporadically in the medical literature.

**Case presentation:**

We report here a case of simultaneous myopericarditis and pulmonary embolism in a 30-year-old man with no medical history. The patient was not immunocompromised. We discuss the possible role of acute cytomegalovirus infection in the induction of vascular damage and review relevant cases in the literature.

**Conclusion:**

Thrombosis in patients with acute cytomegalovirus infection may be more frequent than is generally thought. Physicians need to be aware of the possible association between acute cytomegalovirus and thrombosis in immunocompetent patients, especially in the presence of severe systemic infection, as our case illustrates.

## Background

Primary cytomegalovirus (CMV) infection is common worldwide. The infection usually occurs early in life and then lies dormant. The resulting seroprevalence among adults varies from 40 to 100% depending on the country, socioeconomic conditions and patient age [1-3].

In immunocompetent patients, primary infection is usually asymptomatic or occurs subclinically with a self limited mononucleosis-like syndrome. Prolonged fever, myalgia, headache, cervical lymphadenopathy, splenomegaly, rash and nonspecific constitutional symptoms are common and may persist for weeks [4]. Very occasionally, CMV can cause tissue-invasive end-organ damage and complications including pneumonia [5], myocarditis [6], hemolytic anemia [7], retinitis [8], colitis [9] and hepatitis [10]. Central nervous system involvement with meningitis, encephalitis and Guillain-Barré syndrome may also be observed albeit rarely [11,12].

More recently, primary CMV infection has been associated with venous thrombosis. To date, the medical literature counts close to 100 articles on this association, mainly in case reports and additionally in several small series of patients [13]. Thrombosis has been historically considered as an extremely rare complication of acute CMV infection. However, two recent studies found incidences ranging from 6.4% and 7.9%, suggesting that thrombosis in patients with acute CMV infection may be more common than previously thought [14,15].

We describe here a severe acute CMV infection with myopericarditis and pulmonary embolism. We also review relevant cases in the literature to alert physicians on the possibility of thrombotic events in CMV-infected immunocompetent individuals. We also discuss mechanisms that may explain the role of CMV in thrombosis.

## Case presentation

**Figure 1:**
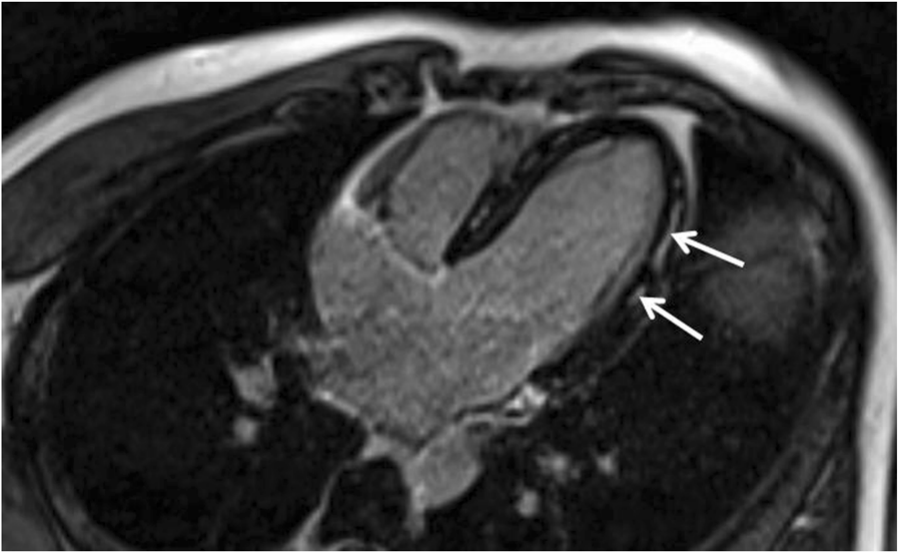
**Typical magnetic resonance imaging image of myocarditis.** This figure shows a magnetic resonance imaging four chamber cardiac view. Mid-wall late gadolinium enhancement of the lateral wall is visible (arrows). This is a common MRI aspect of myocarditis.

A 30-year-old caucasian man was admitted to the cardiac intensive care unit on 27 February 2010 for a two-day history of left precordial chest pain. The patient had no particular medical history other than an episode of viral gastroenteritis that resolved favorably three weeks before his hospitalization. His temperature was 38°C (100.4°F), heart rate 86 beats per minute (BPM), and blood pressure 122/86 mmHg at admission. He described typical pericarditis symptoms with substernal and precordial chest pain relieved by sitting up and bending forward and worsened by lying down and breathing in. There was no radiating pain and physical examination was normal without friction rub. Other symptoms included fatigue and anxiety. White blood cells (WBC) count was 10,250 cells/mm3 with 7,240 neutrophils/mm3 and 1,410 lymphocytes/mm3, hemoglobin was 153.0 g/L with a platelet count of 167,000/mm3. His C-reactive protein level was 33 mg/L (normal < 3 mg/l). Cardiac enzyme tests showed a highly elevated level of troponin Ic at 3.51 μg/L. Alanine aminotransferase (ALT) was at 50 U/L, aspartate aminotransferase (AST) at 202 U/L and creatine kinase (CK) at 2,275 U/L. The electrocardiogram showed a 12-lead diffuse, concave ST segment elevation without low-voltage QRS complexes. Transthoracic echocardiography found a normal left ventricular volume with no kinetic disorder or pericardial effusion. Magnetic resonance imaging (MRI) confirmed myopericarditis with a hyper-intense signal on the MRI late gadolinium enhancement (Figure 1). The chest pain regressed rapidly, the troponin Ic level decreased and the patient was discharged to home four days later with a medical treatment comprising a combination of colchicine 1 mg per day and acetylsalicylic acid 3 g per day for one month.

Three days later, the patient remained febrile (40°C) and presented with a painful right chest with dyspnea. He was readmitted to the cardiac intensive care unit. His temperature was 37.4°C (99.32°F), his heart rate 74 BPM and blood pressure 134/78 mmHg. WBC count was 14,190 cells/mm3, with 3,830 neutrophils/mm3 and 8,370 lymphocytes/mm3 with 2% of the lymphocytes presenting a large hyperbasophilic form. The C-reactive protein level was 100 mg/L. Cardiac enzymes tests showed a normalization of troponin Ic < 0.01 μg/L and CK level of 58 U/L but liver function tests revealed an ALT level of 299 U/L and an AST level of 133 U/L. The electrocardiogram showed the disappearance of ST segment elevation without PR segment depression and the echocardiography was unchanged. Bloody sputum suggested a pulmonary embolism. Chest x-ray showed no opacity on the pulmonary field and the diagnosis was confirmed with a computed tomography (CT) scan showing a bilateral pulmonary embolism of moderate severity with signs of pulmonary infarction. Treatment with intravenous heparin was initiated. Because of the mononucleosis-like biological syndrome, hepatitis virus serology was performed. The results of serological tests for human immunodeficiency virus (HIV) enzyme-linked immunosorbent assay (ELISA), hepatitis A virus IgM, hepatitis B surface antigen, hepatitis C virus, Epstein-Barr virus and toxoplasma IgM antibodies were all negative. For CMV however, ELISA tests were strongly positive for IgM and equivocal for IgG on the first tests performed 7 days after hospital admission. Also at Day 7, quantitative CMV deoxyribonucleic acid (DNA) polymerase chain reaction (PCR) was positive with 2050 CMV DNA copies/mL (ARGENE CMV R-gene™, Biomerieux, France). Of note: CMV PP65 antigen was negative at Day 14. The evolution of the CMV tests is presented in Table 1.

**Table 1:** Evolution of cytomegalovirus tests

	**Day 7 after admission**	**Day 14 after admission**	**Day 28 after admission**
Elisa (CMV Axsym-Abbott)			
Ig M (negative: ratio < 0.4)	1.003	5.852	7.667
Ig G (negative: level < 15UA/ml)	15.2	51.3	165.7
PP65 antigen	-	Negative	-
CMV DNA real-time PCR (copies/mL) (detection range: 150 copies/ml)	2050	7000	<150

Elisa: enzyme-linked immunosorbent assay.

CMV: cytomegalovirus.

Ig: Immunoglobulin.

DNA: deoxyribonucleic acid.

PCR: polymerase chain reaction.

The fever disappeared 5 days after the second admission with no anti-CMV therapy. The patient presented no further chest pain. He felt very tired for two months. Hepatitis was at its maximum 10 days after the second admission but liver tests returned to normal after one month. Heparin was replaced by six months of fluindione. The coagulation work-up included prothrombin time and tests for activated protein C resistance, antithrombin activity, and proteins C and S, as well as an assay for anticardiolipin and anti-beta 2 glycoprotein-1 antibodies. All results were normal.

## Discussion

Acute CMV infection in immunocompetent patients is common worldwide but symptoms remain subclinical most of the time. Rarely, patients may develop a severe infection that manifests with multiple organ involvement and marked constitutional symptoms. Serious CMV infection is more well-known and more frequently described in immunocompromised patients, including in particular transplantation recipients and people with untreated acquired immunodeficiency syndrome (AIDS), where CMV disease is a common cause of death. We describe here an immunocompetent patient with pulmonary embolism and myopericarditis, two rare and severe CMV infection complications.

Pericarditis and myocarditis in immunocompetent patients with acute CMV have been described for years now in the literature [16-18]. CMV has a wide tissue tropism and is able to infect multiple organs including the heart. However, clinically significant cardiac disease is rarely reported overall, although we note an increase of such case-reports in the literature in recent years [19-23] and think that the association may be under-diagnosed currently.

Monitoring of serum antibody levels, which provides a picture of viral pericarditis, is often not performed despite the recommendation to do so in European Society of Cardiology (ESC) guidelines on pericardial diseases [24]. Indeed, an association between CMV and myocarditis was described in a study of more than 1100 patients with active and borderline myocarditis [25]. In that study, PCR of heart samples revealed CMV DNA in 3% of the patients. In another population-based study of immunocompetent patients with fatal myocarditis, CMV DNA was the most common single finding: viral genome was detected by PCR in the cardiac samples of 15 patients (38%), and in 67% of the patients for whom PCR was CMV-positive, in situ hybridization revealed viral DNA in cardiomyocytes [6]. We believe that the development or the aggravation of an acute hepatitis after an obvious clinical improvement of myocarditis and a decrease in cardiac necrosis markers, as noted in several reports, could be suggestive of an acute CMV infection [19,22].

Also, chest pain and heart failure are not obligatory clinical features of myopericarditis. In a series of 115 hospitalized immunocompetent patients with diagnosis of primary CMV infection, the authors found two cases of pericarditis for which the diagnosis was established as a fortuitous finding on routine electrocardiograms in patients without chest pain [26]. Another case report presented an immunocompetent patient with no signs or symptoms of cardiac inflammation. Myocarditis was incidentally diagnosed on trans-thoracic echocardiography, which was undertaken to search for an infectious endocarditis in the setting of a prolonged fever [21].

In recent case-reports, the diagnosis of myocarditis was aided by the presence of clinical signs of pericarditis and cardiac investigations including myocardial necrosis markers and echocardiography [19,22,23]. In our case, the suspicion of heart involvement led us to perform an MRI that confirmed the diagnosis of myopericarditis. The diagnosis of acute CMV infection was confirmed by antibodies in the plasma and the evolution of CMV viral load. Of interest: CMV PP65 antigenemia was negative for our patient. This may be explained by a lesser sensitivity for CMV antigenemia compared to CMV PCR assay, as has been described in the literature, albeit generally in the setting of solid organ or bone marrow transplant patients [27,28]. It may also be explained by the fact that antigenemia was performed only once at Day14; thus it is possible that antigenemia was not yet positive or had already disappeared.

The association of CMV infection and vascular thrombosis was first described in immunocompromised patients, especially in AIDS cases or after transplantation [29-31]. In immunocompetent patients, CMV infection was first described as a risk for coronary thrombotic events after stent placement [32,33]. CMV-associated thrombosis was later reported sporadically in immunocompetent adults with no medical history only in case reports or in small series [13]. However, in two recent studies, the incidence of thrombosis among patients with acute CMV infection was reported to range from 6.4 to 7.9%, suggesting that it may not be so rare after all [15,34]. In a prospective study comparing 187 hospitalized patients with deep vein thrombosis and/or pulmonary embolism to 187 controls without thrombosis, the incidence of acute CMV infection was 9.1% for the former compared to 1.6% for the latter (odds ratio = 6.12; p = 0.016) [35]. In another recent case–control study on 397 consecutive patients with suspected deep vein thrombosis, five were positive for CMV DNA whereas none were in the control group [36]. In that study, all five of the CMV-positive patients were women, below 37 years of age, with another acquired risk for venous thrombosis.

Several hypotheses have been forwarded for the role of CMV in inducing vascular damage. Squizzato et al. performed a literature review focused on the effects of CMV infection on coagulation [37]. CMV probably plays a different role in inducing arterial or venous thrombosis. CMV may induce transient antiphospholipid antibody production [38] and certain strains may also express constitutively phosphatidylserine (PS)-like procoagulant activity. Sutherland et al. demonstrated that tissue factor (TF) antigen, a coagulation initiator, could be identified on CMV using monoclonal antibodies with flow cytometry and electron microscopy [39]. However these properties were not unique for CMV; they were also pertinent for other herpes viruses. Moreover, CMV causes membrane perturbation when it infects endothelial cells and activates a pro-coagulation state. Von Willebrand factor (vWf) has been shown to be released during CMV infection [40], and, among 39 patients with recent kidney transplantation and acute CMV infection, increased vWf and soluble vascular cell adhesion molecule 1 (sVCAM-1) in the peripheral blood were correlated with viral infection [41].

Regarding the development of atherosclerosis and restenosis, smooth muscle cell proliferation and migration are crucial events. It has been suggested that one of the immediate early gene products of CMV, IE84, binds to and inhibits P53 transcriptional activity, thus inhibiting P53-mediated apoptosis, which in turn permits smooth muscle cell proliferation [42,43]. Moreover, CMV infection increases PDGF b-receptor expression, which modulates smooth muscle cell migration [44]. Also, CMV infection may contribute to vascular effects in atherosclerotic plaque inflammation by increasing levels of several cytokines with inflammatory properties [45]. Especially, a modest increase in interleukin (IL) 6 is observed in infected endothelial cells [46]. CMV infection of endothelial cells also increases the expression of CD40 [47], a protein present on atheroma-associated cells and possibly involved in a number of processes responsible for lesion progression and plaque destabilization [48].

Our case-report underlines the interest of CMV serology in the presence of viral myopericarditis. This complication is infrequently reported in the literature but needs to be kept in mind as it may lead to disproportionate aggression to the myocardium and clinical heart failure. Our experience also illustrates the interest of MRI in the diagnosis of myocarditis. Furthermore, the complication may indicate a serious acute CMV infection capable of provoking other serious complications such as vascular thrombosis and pulmonary embolism.

## Conclusion

In conclusion, to our knowledge, the present case is the first to report acute CMV myopericarditis appearing with a thrombotic pulmonary embolism. Physicians must keep this association between severe CMV infection and vascular thrombosis in mind to better implement preventative measures.

## Consent

Written informed consent was obtained from the patient for publication of this Case Report and any accompanying images. A copy of the written consent is available for review by the Editor-in-Chief of this journal.

## Competing interests

The authors declare that they have no competing interests.

## Authors’ contributions

YMV conceived of the Case Report. AD carried out the immunoassays and CMV viral loads, participated in the interpretation and helped to draft the manuscript. LB carried out magnetic resonance imaging, participated in its interpretation and helped to draft the manuscript. NV participated in magnetic resonance imaging interpretation and helped to draft the manuscript. FR participated in magnetic resonance imaging interpretation and helped to draft the manuscript. VD helped to draft the manuscript. PA detected the case-report and oversaw this manuscript. All authors meet the criteria for authorship, including acceptance of responsibility for the scientific content of the article. All authors had access to the data and a role in writing. All authors read and approved the final manuscript.

## References

[B1] Bate SL Dollard SC Cannon MJ Cytomegalovirus seroprevalence in the United States: the national health and nutrition examination surveys, 1988–2004 Clin Infect Dis 2010 50 11 1439 1447 10.1086/652438 20426575 PMC11000537

[B2] Boeckh M Geballe AP Cytomegalovirus: pathogen, paradigm, and puzzle J Clin Invest 2011 121 5 1673 1680 10.1172/JCI45449 21659716 PMC3083799

[B3] Hyde TB Schmid DS Cannon MJ Cytomegalovirus seroconversion rates and risk factors: implications for congenital CMV Rev Med Virol 2010 20 5 311 326 10.1002/rmv.659 20645278

[B4] Wreghitt TG Teare EL Sule O Devi R Rice P Cytomegalovirus infection in immunocompetent patients Clin Infect Dis 2003 37 12 1603 1606 10.1086/379711 14689339

[B5] Klemola E Von Essen R Henle G Henle W Infectious-mononucleosis-like disease with negative heterophil agglutination test. Clinical features in relation to Epstein-Barr virus and cytomegalovirus antibodies J Infect Dis 1970 121 6 608 614 10.1093/infdis/121.6.608 4316146

[B6] Kyto V Vuorinen T Saukko P Lautenschlager I Lignitz E Saraste A Voipio-Pulkki LM Cytomegalovirus infection of the heart is common in patients with fatal myocarditis Clin Infect Dis 2005 40 5 683 688 10.1086/427804 15714413

[B7] Chanarin I Walford DM Thrombocytopenic purpura in cytomegalovirus mononucleosis Lancet 1973 2 7823 238 239 4124426 10.1016/s0140-6736(73)93138-3

[B8] Stewart MW Bolling JP Mendez JC Cytomegalovirus retinitis in an immunocompetent patient Arch Ophthalmol 2005 123 4 572 574 10.1001/archopht.123.4.572 15824240

[B9] Rafailidis PI Mourtzoukou EG Varbobitis IC Falagas ME Severe cytomegalovirus infection in apparently immunocompetent patients: a systematic review Virol J 2008 5 47 10.1186/1743-422X-5-47 18371229 PMC2289809

[B10] Tajiri H Kozaiwa K Tanaka-Taya K Tada K Takeshima T Yamanishi K Okada S Cytomegalovirus hepatitis confirmed by in situ hybridization in 3 immunocompetent infants Scand J Infect Dis 2001 33 10 790 793 10.1080/003655401317074707 11728056

[B11] Schmitz H Enders G Cytomegalovirus as a frequent cause of Guillain-Barre syndrome J Med Virol 1977 1 1 21 27 10.1002/jmv.1890010105 204737

[B12] Eddleston M Peacock S Juniper M Warrell DA Severe cytomegalovirus infection in immunocompetent patients Clin Infect Dis 1997 24 1 52 56 10.1093/clinids/24.1.52 8994755

[B13] Abgueguen P Delbos V Chennebault JM Payan C Pichard E Vascular thrombosis and acute cytomegalovirus infection in immunocompetent patients: report of 2 cases and literature review Clin Infect Dis 2003 36 11 E134 E139 10.1086/374664 12766855

[B14] Justo D Finn T Atzmony L Guy N Steinvil A Thrombosis associated with acute cytomegalovirus infection: a meta-analysis Eur J Intern Med 2011 22 2 195 199 10.1016/j.ejim.2010.11.006 21402253

[B15] Abgueguen P Delbos V Ducancelle A Jomaa S Fanello S Pichard E Venous thrombosis in immunocompetent patients with acute cytomegalovirus infection: a complication that may be underestimated Clin Microbiol Infect 2010 16 7 851 854 10.1111/j.1469-0691.2009.03022.x 19686279

[B16] Rasanen V Saikku P [Cytomegalovirus hepatitis and pericarditis] Duodecim 1968 84 4 270 273 4299511

[B17] Sterner G Agell BO Wahren B Espmark A Acquired cytomegalovirus infection in older children and adults. A clinical study of hospitalized patients Scand J Infect Dis 1970 2 2 95 103 4329177 10.3109/inf.1970.2.issue-2.04

[B18] Wilson RS Morris TH Rees JR Cytomegalovirus myocarditis Br Heart J 1972 34 8 865 868 10.1136/hrt.34.8.865 4341796 PMC486996

[B19] Vanstechelman F Vandekerckhove H Cytomegalovirus myocarditis in an immunocompetent patient Acta Cardiol 2012 67 2 257 260 22641988 10.1080/ac.67.2.2154221

[B20] Baumgratz JF Vila JH Silva JP Fonseca L Rodrigues EA Knobel E Cardiogenic shock due to cytomegalovirus myocarditis: successful clinical treatment Rev Bras Cir Cardiovasc 2010 25 2 149 153 10.1590/S0102-76382010000200004 20802904

[B21] Roubille C Brunel AS Gahide G Vernhet Kovacsik H Le Quellec A Cytomegalovirus (CMV) and acute myocarditis in an immunocompetent patient Intern Med 2010 49 2 131 133 10.2169/internalmedicine.49.2313 20075576

[B22] Fernández-Ruiz M Muñoz-Codoceo C López-Medrano F Faré-García R Carbonell-Porras A Garfia-Castillo C Muñoz-Gómez R Aguado-García JM Cytomegalovirus myopericarditis and hepatitis in an immunocompetent adult: successful treatment with oral valganciclovir Intern Med 2008 47 22 1963 1966 10.2169/internalmedicine.47.1480 19015608

[B23] Zubiaurre L Zapata E Bujanda L Castillo M Oyarzabal I Gutiérrez-Stampa MA Cosme A Cytomegalovirus hepatitis and myopericarditis World J Gastroenterol 2007 13 4 647 648 17278238 10.3748/wjg.v13.i4.647 PMC4065994

[B24] Maisch B Seferovic PM Ristic AD Erbel R Rienmuller R Adler Y Tomkowski WZ Thiene G Yacoub MH [Guidelines on the diagnosis and management of pericardial diseases. Executive summary] Rev Esp Cardiol 2004 57 11 1090 1114 10.1016/S0300-8932(04)77245-0 15544758

[B25] Hufnagel G Pankuweit S Richter A Schonian U Maisch B The European Study of Epidemiology and Treatment of Cardiac Inflammatory Diseases (ESETCID). First epidemiological results Herz 2000 25 3 279 285 10.1007/s000590050021 10904853

[B26] Bonnet F Morlat P Neau D Viallard JF Ragnaud JM Dupon M Legendre P Imbert Y Lifermann F Le Bras M Beylot J Longy-Boursier M [Hematologic and immunologic manifestations of primary cytomegalovirus infections in non-immunocompromised hospitalized adults] Rev Med Interne 2000 21 7 586 594 10.1016/S0248-8663(00)80003-X 10942974

[B27] Nitsche A Oswald O Steuer N Schetelig J Radonic A Thulke S Siegert W Quantitative real-time PCR compared with pp 65 antigen detection for cytomegalovirus (CMV) in 1122 blood specimens from 77 patients after allogeneic stem cell transplantation: which test better predicts CMV disease development? Clin Chem 2003 49 10 1683 1685 10.1373/49.10.1683 14500600

[B28] Leruez-Ville M Ouachee M Delarue R Sauget AS Blanche S Buzyn A Rouzioux C Monitoring cytomegalovirus infection in adult and pediatric bone marrow transplant recipients by a real-time PCR assay performed with blood plasma J Clin Microbiol 2003 41 5 2040 2046 10.1128/JCM.41.5.2040-2046.2003 12734246 PMC154722

[B29] Sullivan PS Dworkin MS Jones JL Hooper WC Epidemiology of thrombosis in HIV-infected individuals. The adult/adolescent spectrum of HIV disease project AIDS 2000 14 3 321 324 10.1097/00002030-200002180-00015 10716509

[B30] Jenkins RE Peters BS Pinching AJ Thromboembolic disease in AIDS is associated with cytomegalovirus disease AIDS 1991 5 12 1540 1542 10.1097/00002030-199112000-00025 1667576

[B31] Oh CK Pelletier SJ Sawyer RG Dacus AR McCullough CS Pruett TL Sanfey HA Uni- and multi-variate analysis of risk factors for early and late hepatic artery thrombosis after liver transplantation Transplantation 2001 71 6 767 772 10.1097/00007890-200103270-00014 11330540

[B32] Zhou YF Leon MB Waclawiw MA Popma JJ Yu ZX Finkel T Epstein SE Association between prior cytomegalovirus infection and the risk of restenosis after coronary atherectomy N Engl J Med 1996 335 9 624 630 10.1056/NEJM199608293350903 8687516

[B33] Neumann FJ Kastrati A Miethke T Pogatsa-Murray G Seyfarth M Schomig A Previous cytomegalovirus infection and risk of coronary thrombotic events after stent placement Circulation 2000 101 1 11 13 10.1161/01.CIR.101.1.11 10618297

[B34] Atzmony L Halutz O Avidor B Finn T Zimmerman O Steinvil A Zeltser D Giladi M Justo D Incidence of cytomegalovirus-associated thrombosis and its risk factors: a case–control study Thromb Res 2010 126 6 e439 e443 10.1016/j.thromres.2010.09.006 20926120

[B35] Schimanski SLB Luxembourg B Rochon J Seifried E Lindhoff-Last E Schambeck CM Cytomegalovirus infection is associated with venous thromboembolism - a case control study J Thromb Haemost 2009 7 Suppl 2 PP-MO-295

[B36] Tichelaar VY Sprenger HG Makelburg AB Niesters BG Kluin-Nelemans HC Lijfering WM Active cytomegalovirus infection in patients with acute venous thrombosis: a case–control study Am J Hematol 2011 86 6 510 512 10.1002/ajh.22006 21509792

[B37] Squizzato A Gerdes VE Buller HR Effects of human cytomegalovirus infection on the coagulation system Thromb Haemost 2005 93 3 403 410 15735787 10.1160/TH04-08-0523

[B38] Delbos V Abgueguen P Chennebault JM Fanello S Pichard E Acute cytomegalovirus infection and venous thrombosis: role of antiphospholipid antibodies J Infect 2007 54 1 e47 e50 10.1016/j.jinf.2006.03.031 16701900

[B39] Sutherland MR Raynor CM Leenknegt H Wright JF Pryzdial EL Coagulation initiated on herpesviruses Proc Natl Acad Sci U S A 1997 94 25 13510 13514 10.1073/pnas.94.25.13510 9391056 PMC28336

[B40] Bruggeman CA Debie WH Muller AD Schutte B van Dam-Mieras MC Cytomegalovirus alters the von Willebrand factor content in human endothelial cells Thromb Haemost 1988 59 2 264 268 2838925

[B41] The TH Kas-Deelen AM de Maar EF Driessen C Harmsen MC van Son WJ Cellular and humoral parameters for vascular damage in blood during cytomegalovirus infections Transplant Proc 2001 33 1–2 1813 11267524 10.1016/s0041-1345(00)02692-0

[B42] Speir E Modali R Huang ES Leon MB Shawl F Finkel T Epstein SE Potential role of human cytomegalovirus and p53 interaction in coronary restenosis Science 1994 265 5170 391 394 10.1126/science.8023160 8023160

[B43] Tanaka K Zou JP Takeda K Ferrans VJ Sandford GR Johnson TM Finkel T Epstein SE Effects of human cytomegalovirus immediate-early proteins on p53-mediated apoptosis in coronary artery smooth muscle cells Circulation 1999 99 13 1656 1659 10.1161/01.CIR.99.13.1656 10190872

[B44] Zhou YF Yu ZX Wanishsawad C Shou M Epstein SE The immediate early gene products of human cytomegalovirus increase vascular smooth muscle cell migration, proliferation, and expression of PDGF beta-receptor Biochem Biophys Res Commun 1999 256 3 608 613 10.1006/bbrc.1999.0387 10080946

[B45] Zhou YF Shou M Guetta E Guzman R Unger EF Yu ZX Zhang J Finkel T Epstein SE Cytomegalovirus infection of rats increases the neointimal response to vascular injury without consistent evidence of direct infection of the vascular wall Circulation 1999 100 14 1569 1575 10.1161/01.CIR.100.14.1569 10510062

[B46] Visseren FL Verkerk MS Bouter KP Diepersloot RJ Erkelens DW Interleukin-6 production by endothelial cells after infection with influenza virus and cytomegalovirus J Lab Clin Med 1999 134 6 623 630 10.1016/S0022-2143(99)90103-8 10595791

[B47] Mach F Schonbeck U Libby P CD40 signaling in vascular cells: a key role in atherosclerosis? Atherosclerosis 1998 137 Suppl S89 S95 9694547 10.1016/s0021-9150(97)00309-2

[B48] Schonbeck U Libby P CD40 signaling and plaque instability Circ Res 2001 89 12 1092 1103 10.1161/hh2401.101272 11739273

